# Current Approaches to the Management of Acute Thoracolumbar Disc Extrusion in Dogs

**DOI:** 10.3389/fvets.2020.00610

**Published:** 2020-09-03

**Authors:** Sarah A. Moore, Andrea Tipold, Natasha J. Olby, Veronica Stein, Nicolas Granger, Canine Spinal Cord Injury Consortium (CANSORT SCI), Sarah A. Moore

**Affiliations:** Author Affiliations: DACVIM-Neurology Associate Professor, Neurology and Neurosurgery Department of Veterinary Clinical Sciences The Ohio State University College of Veterinary Medicine Columbus Ohio, 43026; DACVIM Neurology Professor of Neurology/Neurosurgery; Distinguished Chair of Gerontology Department of Clinical Sciences North Carolina State University College of Veterinary Medicine Raleigh, NC 27607; DACVIM-Neurology Professor, Helen McWhorter Chair, and Head Department of Small Animal Clinical Sciences College of Veterinary Medicine and Biomedical Sciences Texas A&M University College Station, TX 77843-4474; DAVCIM (Neurology) Assistant Professor of Neurology Purdue University College of Veterinary Medicine Department of Veterinary Clinical Sciences West Lafayette, IN 47907; Professor Neurology & Neurosurgery; Professor in Small Animal Clinical Sciences College of Veterinary Medicine Texas A&M University TX 77843; ACVIM - Neurology Professor and Service Head, Neurology and Neurosurgery Department of Veterinary Clinical Sciences College of Veterinary Medicine The Ohio State University Columbus, OH 43210-1089; Department of Clinical Sciences Colorado State University Fort Collins, CO 80523-1678; Department of Clinical Science and Services Royal Veterinary College, Hawkshead Lane Hatfield, Hertfordshire, UK; The Royal Veterinary College, University of London, Hawkshead Lane, Hatfield, Hertfordshire, United Kingdom & CVS Referrals, Bristol Veterinary Specialists at Highcroft, Bristol, United Kingdom; Institute of Veterinary Pathology Faculty of Veterinary Medicine Leipzig University D-04103 Leipzig Germany; Division of Clinical Neurology Department for Clinical Veterinary Medicine Vetsuisse Faculty University of Bern CH-3012 Bern Switzerland; Dept Small Animal Medicine and Surgery University of Veterinary Medicine Hannover D-30559 HANNOVER Germany/Europe; Neurology and Neurosurgery, Department of Veterinary Medicine and Surgery University of Missouri MU Veterinary Health Center University of Missouri, Columbia, MO 65211; Dept of Small Animal Medicine and Surgery University of Veterinary Medicine Hannover D-30559 HANNOVER Germany/Europe; ^1^Department of Veterinary Clinical Sciences, The Ohio State University College of Veterinary Medicine, Columbus, OH, United States; ^2^Department Small Animal Medicine and Surgery, University of Veterinary Medicine Hannover, Hanover, Germany; ^3^Department of Clinical Sciences, North Carolina State University College of Veterinary Medicine, Raleigh, NC, United States; ^4^Division of Clinical Neurology, Department for Clinical Veterinary Medicine, Vetsuisse Faculty, University of Bern, Bern, Switzerland; ^5^The Royal Veterinary College, University of London, Hatfield, United Kingdom; ^6^CVS Referrals, Bristol Veterinary Specialists at Highcroft, Bristol, United Kingdom; ^7^The Canine Spinal Cord Injury Consortium, Hanover, Germany

**Keywords:** dog, interveterbral disc, intervertebral disc disease, hemilaminectomy, spinal cord injury, hansen type I

## Abstract

Intervertebral disc extrusion (IVDE) is one of the most common neurologic problems encountered in veterinary clinical practice. The purpose of this manuscript is to provide an overview of the literature related to treatment of acute canine thoracolumbar IVDE to help construct a framework for standard care of acute canine thoracolumbar IVDE where sufficient evidence exists and to highlight opportunities for future prospective veterinary clinical research useful to strengthen care recommendations in areas where evidence is low or non-existent. While there exist a number of gaps in the veterinary literature with respect to standards of care for dogs with acute thoracolumbar IVDE, recommendations for standard care can be made in some areas, particularly with respect to surgical decompression where the currently available evidence supports that surgery should be recommended for dogs with nonambulatory paraparesis or worse. While additional information is needed about the influence on timing of decompression on outcome in dogs that are deep pain negative for longer than 48 h duration, there is no evidence to support treatment of the 48 h time point as a cut off beyond which it becomes impossible for dogs to achieve locomotor recovery. Surgical decompression is best accomplished by either hemilaminectomy or mini-hemilaminectomy and fenestration of, at a minimum, the acutely ruptured disc. Adjacent discs easily accessed by way of the same approach should be considered for fenestration given the evidence that this substantially reduces future herniation at fenestrated sites. Currently available neuroprotective strategies such as high does MPSS and PEG are not recommended due to lack of demonstrated treatment effect in randomized controlled trials, although the role of anti-inflammatory steroids as a protective strategy against progressive myelomalacia and the question of whether anti-inflammatory steroids or NSAIDs provide superior medical therapy require further evaluation.

## Introduction

Intervertebral disc extrusion (IVDE) is one of the most common neurologic problems encountered in veterinary clinical practice (1). Dogs with acute IVDE present with a spectrum of neurologic abnormalities caused by a combination of compression and contusion to the spinal cord via sudden extrusion of degenerated and calcified nucleus pulposus of the intervertebral disc through the annulus fibrosus and into the vertebral canal (2). Severity of clinical injury spans a continuum from paraspinal hyperesthesia up to paraplegia with loss of deep pain perception, where these patients with loss of deep pain perception are often termed “deep pain negative.” Injury grades are typically described as summarized in Table 1 (3, 4). Treatment recommendations for an individual dog with IVDE are based on a combination of factors, accounting for the aforementioned severity of neurologic signs presented, availability of specialty care in a geographical area, and preferences and financial limitations of the owner. Available treatment options include medical management (often termed “conservative therapy”), consisting of strict activity restriction, physiotherapy, analgesics and anti-inflammatory medications; or surgical decompression of the spinal cord to remove herniated material from the vertebral canal, followed by similar activity restriction, bladder management if needed, and pain management recommendations. The evidence available in the veterinary literature to guide practitioners in recommending one therapeutic approach over another in dogs with IVDE is relatively low as most published veterinary studies are either retrospective in nature or prospective case series. Some randomized clinical trials and two recent systematic review and meta-analyses are available to inform care recommendations; however, this small database of strong clinical evidence has resulted in a lack of uniform, science-based guidelines for the management of acute canine thoracolumbar IVDE.

**Table 1:** Modified Frankel scale used to describe the degree of neurologic impairment for dogs with intervertebral disc herniation.

**Grade**	**Clinical signs**
0	Paraplegia with absent superficial and deep pain sensation (also termed “deep pain negative” throughout the literature); there is an absence of behavioral response (e.g., vocalizing or orienting movement of the head toward stimulus) when clamping of a hemostat or other instrument to the skin of the limb/paw (superficial) and when applying the same stimulus while clamping the bone of the digit (deep). There is no observable voluntary movement of the hind limbs
1	Paraplegia with absent superficial but intact deep pain sensation; behavioral response (e.g., vocalizing or orienting movement of the head toward stimulus) is absent when clamping the skin (superficial) but present when clamping the bone of the digit (deep). There is no observable voluntary movement of the hind limbs. Note that some studies group this subset of dogs with either grade 0, or grade 2 depending on study design
2	Paraplegia with intact superficial and deep pain sensation (also termed “deep pain positive” throughout the literature); there is presence of a behavioral response (e.g., vocalizing or orienting movement of the head toward stimulus) to both superficial and deep noxious stimuli. There is no observable voluntary movement of the hind limbs
3	Non-ambulatory paraparesis; there is movement of one or both hind limbs, but the animal is unable to take 10 consecutive unassisted weight-bearing steps
4	Ambulatory paraparesis; the animal can take 10 consecutive unassisted weight-bearing steps with the hind limbs but displays an ataxic or paretic gait
5	Paraspinal hyperesthesia only; the animal has a normal hind limb gait but has posture or physical examination findings indicative of paraspinal hyperesthesia
6	Normal

The purpose of this manuscript is to build on previously published studies by incorporating broad historical and contemporaneous clinical data to construct a framework for standard care of acute canine thoracolumbar IVDE where sufficient evidence exists and to highlight opportunities for future prospective veterinary clinical research useful to strengthen care recommendations in areas where evidence is low or non-existent. While a number of distinct clinical presentations of intervertebral disc disease occur in dogs, this paper focuses specifically on acute thoracolumbar IVDE, from here on referred to as IVDE, for which the largest body of evidence exists to base treatment recommendations.

## A Historical Perspective

The first description of the clinical presentation of IVDE in dogs is credited to Dexler, who in the late 1800's described a condition of paralysis in dogs caused by compression of the spinal cord from abnormalities of the intervertebral disc that he termed “neoformations” (5). He attributed the neoformations to proliferation of the intervertebral disc, a hypothesis supported by subsequent works that used the term *endochondrosis intervertebralis* to describe the condition. It wasn't until the late 1930's and 1940's that authors began to suggest that these neoformations might in fact be herniation of the intervertebral disc similar to what had been observed in people (6, 7). In 1951 and 1952, Olsson and Hansen (respectively), published in-depth investigations of the syndrome; both supported the fact that these neoformations were in fact disc herniations (5, 8). Hansen's report on the condition further highlighted three breeds with an apparently high risk for the condition: the French bulldog, the dachshund, and the Pekingese (8).

Early diagnosis of IVDE was made by radiography, myelography and pathology. Improvement after conservative treatment was described in individual cases (7). Additional in-depth studies were performed by Hoerlein and published in 1956 and were summarized again after more than 30 years experience (9, 10). Diagnosis evolved over time to become based on physical and neurological examination, radiography, myelography and occasionally tomography. At that time, conservative therapy was recommended for dogs with spinal pain as the only clinical sign, for dogs with a first episode of IVDE and paresis, dogs who had IVDE in combination with other medical disorders, those with paralysis and no conscious perception of deep pain, and those with evidence of progressive myelomalacia (9). Conservative therapy consisted of general good nursing care including proper nutrition, cage rest, ensuring a clean environment, prevention of decubital sores, and bladder and bowel care. Various protocols for glucocorticoid administration were recommended and applied based on coincident clinical research, starting with dexamethasone, and then, in later years, methylprednisolone (11, 12). Physiotherapy, which included limb exercises and swimming, was recommended although a controlled study had not been undertaken at that time. Medications to control pain were recommended, but clinical texts emphasized that pain should not be completely relieved in the outpatient setting due to concern that a completely comfortable patient might undertake excessive movements and resist cage rest.

Hoerlein personally observed 1,184 dogs with IVDE between 1950 and 1975, finding good surgical results in 87% of paraplegic dogs and in 91% of paretic dogs (10). While a true comparative study was never carried out, the rate of functional recovery was described as much lower for cases managed conservatively, where only 22% of paraplegic dogs recovered the ability to ambulate without assistance (13). Additional cases of dogs who ranged from ambulatory paraparetic to paraplegic with deep pain sensation intact managed conservatively and published between 1950 and 1970 suggested that about half of cases recovered the ability to ambulate without surgery. Therefore, surgical treatment was recommended in cases with pain and/or paresis not responding to conservative care and in cases with substantial neurologic deficits where deep pain sensation was preserved with a reported success rate of 75–90% (9, 10, 14).

## Contemporary Questions

While historical evaluation of outcomes associated with surgical decompression in dogs with IVDE suggests that decompression offers improved recovery over conservative management for dogs with severe injuries, no studies have systematically compared outcomes between dogs managed medically and surgically for the condition. Beyond this, additional questions remain in the veterinary neurosurgical community regarding the importance of urgent decompression in deep pain negative dogs; the need for prophylactic fenestration to lessen recurrence of IVDE in dogs at high risk; and the value of neuroprotective strategies and post-operative interventions such as activity restriction and physiotherapy. The following sections discuss the relevant veterinary literature with respect to each area.

## The Evidence for Decompression

### Overview of Current Practices

While only a few contemporary studies exist reporting medical management in dogs with severe neurologic deficits due to IVDE, and no randomized controlled studies compare these to surgical treatment, there is a substantial amount of historical literature from which to draw some basis for comparison (13, 15, 16). One difficulty in evaluating outcomes from early reports of canine IVDE is that patient assessment, diagnostic approach, terminology and description of neurologic deficits, and injury grading differ from more recent literature and make it challenging to draw strong conclusions about neurologic grade at presentation and its relationship to long-term outcome. A summary of reported outcomes for dogs with severe IVDE managed medically and published before 1983 is presented in Table 2, and a recent systematic review and meta-analysis compares results between medical and surgical management for cases published after 1983 (17). Reported outcomes for dogs managed medically after severe (non-ambulatory paraparetic or worse) neurologic injury due to IVDE described in more recent publications range from 50 to 100%, depending on the severity of injury and the study (17–24). Clinicians anecdotally suggest that while recovery of ambulation after surgical vs. medical management in dogs with paraparesis or paraplegia with intact deep pain sensation may ultimately be comparable, recovery is quicker and more complete for dogs that undergo surgical decompression (25). At present, most board-certified neurologists and orthopedic surgeons recommend surgical decompression for dogs who are non-ambulatory paraparetic or worse secondary to IVDE suggesting that this is the current standard of care (26).

**Table 2:** Synthesis of outcome data from dogs managed medically for intervertebral disc herniation and published before 1983.

**Reference**	**Medical management (N)**	**Recovered (%)**
Olsson (5)	21	12 (57)
Hoerlein (9)	62	32 (52)
Funkquist (13)	33	16 (48)
Funkquist (15)	23	14 (61)
Total	139	74 (53)

*Neurologic grade of individual dogs according to the modified Frankel scale at presentation for individual animals is difficult to discern from some papers and these cases represent a heterogenous group of dogs ranging from paraparetic to paraplegic. Wherever possible, based on case description, grade 1 and 5 dogs have been excluded from analysis to assist with comparison to more recent literature. Recovery is defined as return of ambulation based on what is described in the manuscript and only cases with reported post-treatment follow up are included*.

The question of whether or not surgical decompression should be considered standard for dogs with substantially compressive IVDE has been raised and revisited intermittently across the veterinary literature (16, 27, 28). Indeed, several notable studies report that a portion of dogs with substantially compressive IVDE can recover with conservative therapy alone, and that in some cases extruded disc material may even dissipate over time (29–31). Clinicians likely make their current recommendations for surgery based on both the historical literature and having absorbed implications from the field of experimental spinal cord injury suggesting that very early surgical decompression leads to enhanced recovery (32). The drive to perform surgical decompression in the most severe cases may also emanate, in part, from “modern” owner's expectations, our perception of animal welfare in recent decades, and the fact the demonstration of compression on cross-sectional imaging drives an impulse to decompress. Additional influence may also include fairly predictable outcomes with surgery, allowing dog owners to opt for surgery based on concrete numbers; however, no prospective randomized trial has evaluated conservative management *vs*. surgical decompression in dogs with severe IVDE. Currently, the likelihood of this study happening is low, given the lack of clinical equipoise across the veterinary community related to the currently available data indicating the value of decompression.

### Outcome for Severe Injuries (Non-ambulatory Paraparesis or Worse)

While no randomized prospective clinical trials have compared these two treatment options, we can draw some inferences from the existing literature to form a framework for discussing the value of decompressive surgery. In addition to the historical literature available for review, Langerhuus and Miles conducted a systematic review and meta-analysis of dogs with IVDE published between 1983 and 2012 and treated either medically or surgically (17). For dogs with severe injuries (non-ambulatory paraparetic or worse), a statistically greater proportion recovered independent ambulation following decompression *via* hemilaminectomy as compared to conservative treatment. Specifically, 93% of dogs who were non-ambulatory paraparetic or paraplegic with intact deep pain perception recovered ambulation after surgery whereas only 79% of dogs with non-ambulatory paraparesis and 62% of dogs who were paraplegic with intact deep pain perception recovered after conservative treatment alone. For dogs with the most severe injuries, those who were paraplegic and deep pain negative, the recovery rate was 61% after surgical decompression and 10% for those with conservative treatment (although of note here, data from only 25 deep pain negative dogs managed conservatively was available for inclusion). A summary of neurologic outcomes, as reported in this meta-analysis by injury grade is presented in Table 3. The influence of surgical decompression on time to return of independent ambulation (e.g., speed of recovery) is more difficult to assess *via* synthesis of previous studies. Fewer data points are available for inclusion in meta-analysis and preclude analysis of recovery by neurologic grade for all severities except paraplegia with intact deep pain perception; however, for dogs with paraplegia with intact deep pain perception, mean time to recovery of ambulation is significantly shorter after surgical decompression compared with conservative therapy (15 vs. 84 days, respectively) (17).

**Table 3:** Overall percent of dogs achieving neurologic recovery (defined as independent ambulation) based on presenting injury severity, as reported in the literature after 1983 by Langerhuus and others for medical and surgical management of canine thoracolumbar interverterbral disc herniation (IVDH).

**Injury severity**	**Number of cases reported**	**Medical management**	**Surgical decompression**	**References**
Paraplegia with absent deep pain sensation	513	10%	61%	(17)
Paraplegia with intact deep pain sensation	603	62%	93%	(17)
Non-ambulatory paraparesis	354	79%	93%	(17)
Ambulatory paraparesis	152	48–84%	95%	(18, 23, 25)
Paraspinal hyperesthesia only	143	60–100%	97%	(18, 23, 25)

### Limitations of Available Outcome Data for Severe Injuries (Non-ambulatory Paraparesis or Worse)

Taken in its entirety, synthesis of the previously published veterinary literature supports the standard recommendation for surgical decompression in dogs that are non-ambulatory paraparetic or worse, and that surgery speeds recovery of ambulation and overall locomotor outcome in dogs with severe injuries. There are some limitations to the data used to come to this conclusion that must be acknowledged. Since no prospective, randomized studies are available, all synthetized reports comparing outcome incorporate data only from prospective case series and retrospective studies. Additionally, sufficiently detailed outcomes for dogs managed conservatively are available for a relatively small number of published cases, with only 113 cases described in the literature published since 1983 and a similarly small number described before that date. In comparison, >1,500 surgically treated cases and their associated outcomes are described over the same time period. The paucity of published medically managed cases with severe IVDE, while not surprising given current clinical standards, likely confounds comparison of outcomes. Limited publication of medically managed cases probably results from several factors that bias the entirety of the published literature on canine IVDE. First, there is an overall pre-existing clinical inclination toward recommending decompression for dogs that are non-ambulatory paraparetic or worse due to IVDE. In the face of severe clinical signs, clinicians are naturally driven to administer a treatment because they can. Second, most published case series originate from veterinary specialty referral centers where access to advanced care and recommendation for and compliance with decompressive surgery are likely to be higher. Few published cases originate from primary care facilities, where the rate of and approaches toward medical management are likely to be different. Third, data available and used for synthesis of the literature is largely of retrospective nature. Therefore, caution should be used in over interpretation.

### Outcome for Mild Injuries (Paraspinal Hyperesthesia or Ambulatory Paraparesis)

As discussed above, while some population-level analysis of clinical data for dogs with severe injury from IVDE exists to guide treatment recommendations, there is a current gap in the veterinary literature with respect to medical and surgical outcomes for dogs with more mild injuries (those who have paraspinal hyperesthesia or ambulatory paraparesis). Because these dogs were not included in subgroup analysis of outcome in a recent meta-analysis, outcomes for those dogs are summarized in Table 3 from previous literature (18, 23, 25). While some clinicians steer owners toward conservative management in these more mildly affected cases, 12 and 59% of neurologists and orthopedic surgeons (respectively), report routinely recommending surgical decompression for dogs with a first episode of IVDE causing back pain or ambulatory paraparesis (26). The evidence to guide practitioners in these more mildly affected cases is currently lacking as few studies specifically examine outcome for dogs in this context. To the authors' knowledge, no large-scale published comparison exists for dogs these dogs with less severe injuries treated medically *vs*. surgically, although one study reported a 54.7% overall success rate for medical management of thoracolumbar IVDE in a cohort of dogs for which 83% were ambulatory on presentation, and several other studies report surgical outcome for a small number of dogs with mild injuries (23). Hurdles to designing randomized controlled studies for dogs with mild IVDE include the difficulty in obtaining a definitive diagnosis for dogs that are not managed surgically, and the fact that most mildly affected dogs are treated at primary care facilities without referral to a specialist. Albeit more challenging to quantify in dogs, and while recovery of locomotion is of considerable importance to owners of dogs with IVDE, relevant long-term outcomes for dogs with mild injuries might also focus on pain and quality of life measures. Given the fact that a substantial portion of dogs with IVDE present with only pain or mild neurologic deficits, large scale prospective studies could be ethically conducted in this area and represent an opportunity to improve our understanding of best practices in treating this substantial patient population (33–37). Well-powered and informative prospective studies in this area will likely require partnership between specialty referral hospitals and primary care facilities and may require the development of novel clinical assessment tools. In particular, it would be useful to conduct a longitudinal study—from puppy to end of life—recording the environment, health and behavior in breeds at risks of IVDE. Feasibility and utility of generating powerful epidemiological data to answer health questions spanning the life course has been previously demonstrated in dogs, cats, and people (38, 39).

## Surgical Approach

A variety of descriptions of surgical approaches to address canine thoracolumbar IVDE have been previously published. These include hemilaminectomy, mini-hemilaminectomy/pediculectomy, dorsal laminectomy, partial corpectomy, and fenestration of the intervertebral disc with or without concurrent laminectomy for removal of herniated disc material (34, 40–42).

Early studies reported spinal decompression procedures for canine IVDE *via* either dorsal laminectomy or hemilaminectomy. Hoerlein and colleagues described a procedure for hemilaminectomy in detail, whereas Funkquist explored procedures for dorsal laminectomy (15, 43). A study published by Hoerlein in 1978, and using a questionnaire gathering information from 50 participating veterinary surgeons about the use of surgical approaches for treatment of IVDE, suggested that the best surgical results were obtained after hemilaminectomy and fenestration (44). These results were later confirmed in a second prospective but non-randomized study comparing hemilaminectomy to dorsal laminectomy for treatment of thoracolumbar IVDE, where hemilaminectomy was reported to significantly improved the surgeon's ability to retrieve herniated disc material and where this enhanced removal of herniated disc was associated with improved early locomotor recovery (45).

Today, hemilaminectomy with or without removal of the articular processes is most commonly described and represents the current decompressive procedure of choice for veterinary spinal surgeons. A recent survey indicated that 95% percent of veterinary neurologists and surgeons typically perform a hemilaminectomy or mini-hemilaminectomy in this scenario (26). While each disc herniation is different, and requires unique consideration for what constitutes the best surgical approach, efficiency in disc retrieval, reduced opportunity for laminectomy membrane formation, and reduced chance of postoperative neurologic decline likely form the relatively broad surgical consensus for hemilaminectomy in the surgical treatment of routine thoracolumbar IVDE (45–47). Table 4 summarizes the published literature relating to surgical approaches for canine IVDE, including benefits and unique challenges of each approach.

**Table 4:** A summary of published surgical approaches and reported outcomes for canine acute intervertebral disc herniation (IVDH) affecting the thoracolumbar spine.

**Surgical approach**	**Description**	**Advantages**	**Limitations**	**References**
Dorsal laminectomy	Removal of the spinous process and variable portion of the lamina with conservation of the articular processes	• Increased cord exposure compared to hemilaminectomy • Improved access to dorsal compressive lesions	• No access to ventral portion of the vertebral canal for disc removal • Concern for laminectomy scar formation, particularly if more than one consecutive site	(15, 43, 46, 47)
Hemilaminectomy	Removal of half of the vertebral arch, including the lamina, pedicle, and articular process	• Reduced laminectomy scar • Improved access to ventral portion of the spinal canal for disc removal • Improved access for fenestration	• Residual compression is common (clinical significance unclear)	(17, 19, 48–50)
Mini-hemilaminectoy/pediculectomy	Similar to a hemilaminectomy but articular process is spared	• Less invasive than hemilaminectomy • Improved access to ventral portion of the spinal canal for disc removal • Improved access for fenestration	• Residual compression is common (clinical significance unclear)	(41, 51, 52)
Partial corpectomy	Partial removal of thoracic or lumbar adjacent vertebral bodies that support the extruded/protruded disk material inside the vertebral canal	• Allows ventral decompression with minimal spinal cord manipulation • May offer an advantage for chronic and ventrally located disc herniations	• Hemorrhage from the venous sinus is common • Transient post-operative deterioration common • Residual compression is common (clinical significance unclear)	(40, 42)
Fenestration without laminectomy	Mechanical removal of the nucleus pulposus through a window created in the annulus fibrosus	• Less invasive than laminectomy • Good outcome for grade 1 and 2 injuries	• Does not relieve spinal cord compression • Reduced and prolonged recovery with severe injuries	(53–56)

Fenestration of the intervertebral disc without spinal cord decompression has also been historically proposed as a viable option for treatment of IVDE, with the idea re-introduced more recently into the veterinary literature by way of a systematic review presenting outcomes of previously published cases (27, 53, 54). While most articles on fenestration address its role in prophylaxis of recurrent disc extrusion (the arguments for and against that approach are presented below), some authors have suggested that fenestration alone may be useful to facilitate recovery of spinal cord function following IVDE. When first described as a therapeutic intervention for IVDE, the aim of fenestration was, in fact, to reduce intradiscal pressure with the goal of reducing a presumed dynamic lesion of the portion of the disc herniated into the epidural space (56). Dogs with IVDE ranging from pain-only to paraplegia with intact deep pain perception, treated only with lateral fenestration of the intervertebral disc were reported to experience a relatively high recovery rate; however, only a 33% recovery rate was reported for paraplegic deep pain negative dogs as compared to the 50–60% recovery rate typically observed for this group after hemilaminectomy (17, 20, 55). Similar to conservative management in deep pain negative dogs, the number of deep pain negative dogs managed with fenestration alone and published in the literature is quite small. Data also originates almost exclusively from retrospective studies, making outcome evaluation challenging. A recent systematic review suggests that outcome for dogs with mild injuries undergoing fenestration alone could be better than previously suggested; however, a retrospective study of 331 dogs undergoing percutaneous disc ablation without decompression (and thus a procedure similar to fenestration alone) demonstrated a recovery rate of only 38% for dogs with deep pain negative injuries, reinforcing the concept that patients with severe injuries benefit from decompression (27, 57). Currently, <10% of veterinary neurologists and orthopedic surgeons report routinely performing fenestration without concurrent decompression (26). This practice pattern may be influenced by previous work suggesting that 80% of dogs presented with back pain as their only clinical sign still have significant spinal cord compression, and thus logically might benefit from decompression (58). Of those surgeons who perform fenestration alone, most indicate they recommend this approach only for dogs with a presenting complaint of spinal pain alone or spinal pain with minimal neurologic deficits (26). At present, evidence to support fenestration alone as a viable surgical approach for canine IVDE is limited and historical literature supports that decompression of the spinal cord, with or without concurrent fenestration, provides improved recovery for dogs with severe (non-ambulatory paraparetic or worse) IVDE (Table 3). Lacking from the current literature is data on the incidence of postoperative chronic pain when fenestration is used without concurrent decompression.

## The Timing of Decompression

When considering the evidence related to the timing of decompression, two clinically important questions arise. Those center on whether decompression should be performed urgently for dogs with severe injuries, and whether dogs that are deep pain negative for a prolonged duration have a reasonable potential for recovery after surgical decompression.

### Is There Value in Decompressing Dogs Who Have Been “Deep Pain Negative” for an Extended Period?

Early studies, and most veterinary neurosurgery texts, suggest that timing of decompression influences outcome, particularly for dogs who are deep pain negative secondary to IVDE. Early decompression is typically encouraged, with a recommended timeframe ranging from 12 to 48 h, beyond which prognosis is often suggested to worsen significantly. These recommendations originate from several retrospective studies, most of which include very few dogs with an injury duration of 48 h or greater. This sentiment seems to persist in spite of several studies that have shown the contrary, noting good functional recovery in some deep pain negative dogs with extended injury duration (72 h or more). A challenge in drawing firm conclusions on this topic is the small number of published deep pain negative cases with a duration of injury longer than 48 h and a confirmed postoperative outcome. Jeffery et al. recently evaluated the influence of a variety of clinical factors on outcome in 78 deep pain negative dogs using a prospective multicenter cohort study design (59). Similar to previous reports, his group was also unable to find an association between duration of deep pain negative status and outcome in this patient population; however, a relatively small number of dogs were available for inclusion where the duration of onset of locomotor dysfunction and initial evaluation at a referral center was >48 h. Currently, some clinicians treat this 48 h time point prior to referral as a “cliff” or abrupt point beyond which recovery in deep pain negative dogs cannot be achieved. There is certainly no literature to support this interpretation but the influence of prolonged deep pain negative status on recovery rate, and importantly on extent of recovery, is also not clear because the number of published cases in any one study is low. Designing a large-scale randomized controlled trial assessing the influence of duration of deep pain negative status on outcome after surgical decompression would be ethically challenging; however, a systematic review and meta-analysis of previously published cases could yield additional valuable information and may assist with guiding owners regarding prognosis for locomotor recovery and the overall utility of surgical decompression for more chronic cases. Larger scale, prospective longitudinal cohort studies could also be helpful and could leverage existing resources already in use across veterinary referral networks (60).

### How Quickly Should Decompression Occur to Maximize Opportunity for Neurologic Recovery in Dogs With Severe Injuries?

Recommendations for urgent decompression, particularly for dogs who are deep pain negative, likely stem from some of the previously mentioned studies on surgical outcomes as well as from the experimental and human spinal cord injury literature where some studies suggest that early decompression is associated with enhanced locomotor recovery; however, the human clinical literature is mixed with regard to the effect of timing of decompression on outcome. A more recent prospective, multi-institutional cohort study of 888 patients with acute spinal cord injury failed to demonstrate an influence of timing of decompression on outcome across the entire cohort and specifically in the group of ASIA Impairment Scale (AIS)- A individuals, those with a clinical injury severity somewhat analogous to deep pain negative status. Interestingly, this study did show an association between improved locomotor outcome and early decompression (<24 h) in groups of patients with incomplete (paresis or plegia with pain sensation intact) injuries (61). What becomes difficult in terms of comparison between dogs and people is that standard recovery curves differ substantially between the two species. Where the reported outcome for locomotor recovery in deep pain negative dogs ranges from 50 to 60%, the incidence of recovery in people with equivalent injuries is much lower; thus drawing strong direct parallels between recovery curves for the two can be challenging. It is likely that people with sensorimotor complete (deep pain negative) injuries represent a much more “complete” injury in many cases and therefore the ability to influence recovery may be more limited whereas those with less complete injuries may be more amenable to intervention. Additionally, most people presenting with spinal cord injury are polytrauma patients with acute concerns related to hypotension, internal injuries and other co-morbid conditions. Therefore, delay in decompression is often necessary in favor of stabilization of the patient for general anesthesia. In most dogs with IVDE, this is not the case and delay of anesthesia for medical reasons is rarely necessary.

As noted above, a recent study was not able to demonstrate improved neurologic outcome in deep pain negative dogs with early surgical intervention, although most patients included in that study were referred for decompressive surgery within 24 h, therefore potentially confounding the ability to demonstrate associations (59). Interestingly, while another recent retrospective study by Castel et al. also supported the lack of influence of timing of surgery on locomotor recovery, this study did find an association between delay of decompression beyond 12 h and increased risk of progressive myelomalacia, an uncommon but often fatal phenomenon observed almost exclusively in dogs who are deep pain negative secondary to IVDE (62).

Taken in total, the evidence in the veterinary literature supporting the need for emergent/immediate decompression in dogs that are deep pain negative secondary to IVDE is low and there likely exists a subset of dogs with severe spinal cord injury that, due to the severity of their injury, will not improve regardless of speed of intervention; however, the evidence to the contrary is also low and a threshold beyond which outcome may worsen has not been established. While warranting further investigation, an increased risk of myelomalacia with delayed decompression might support the recommendation to undertake decompression in severely injured dogs ideally within 12–24 h. The current literature lacks data specific to dogs that are paraplegic with intact deep pain, as these dogs are often grouped either with non-ambulatory paraparetic dogs, or with paraplegic deep pain negative dogs depending on study design. Specifically, it is not known how many dogs progress from paraplegic or paraplegic and deep pain negative when left briefly untreated, e.g., overnight.

## The Need for Fenestration

Perhaps the most historically controversial issue in veterinary neurosurgery centers on the question of prophylactic fenestration of the intervertebral disc both at sites of current extrusion and at distant sites. The concept of intervertebral disc fenestration has been advanced by some veterinary spinal surgeons as a preventative measure which can be taken at the time of decompressive surgery to reduce future extrusion of disc material at sites adjacent to those affected at the time of the original procedure (34). Typically, fenestration of intervertebral discs between T11 and L4 are approached dorsolaterally or laterally at the time of surgery for a extruded disc, and a window is made into the annulus fibrosus with various means employed to evacuate any degenerated nucleus pulposus *in situ* (63–68). Fenestration of the L4-5 and L5-6 spaces is not typically performed due to concern for injury of the nerve roots essential for weight bearing at that location (69). In the context of acute canine IVDE, fenestration is performed “always” or “most of the time” by 69% of board-certified neurologists and 36% of board-certified surgeons (26). Clinicians who do not routinely fenestrate cite concerns including questionable efficacy; prolonged surgical time; complications such as hemorrhage, pneumothorax or nerve root injury; variable success in removal of *in situ* nucleus pulposus; potential for introduction of additional disc material into the vertebral canal; induction or worsening of degenerative changes to non-herniated discs, and the concern for adjacent segment disease (70, 71). Clinicians who do routinely fenestrate cite a recurrence rate as high as 40% for IVDE and the fact that dogs who present for a second bout of surgical IVDE have a rate of euthanasia as high as 44%, often due to financial concerns of the owner (34, 72).

A number of large-scale retrospectives, and two prospective studies have evaluated the effect of fenestration on recurrence of IVDE (34, 36, 66, 69, 72, 73). It is clear from these and other studies that the likelihood of recurrence increases with the number of calcified discs *in situ* present. All studies support the concept that prophylactic fenestration is generally successful in reducing future extrusion of disc material at fenestrated disc spaces and that second disc extrusions, when they occur in dogs that have undergone prophylactic fenestration in conjunction with a history of previous decompressive surgery, are more likely to happen at non-fenestrated sites (34, 73). Results of large-scale contemporary studies evaluating outcome and recurrence after hemilaminectomy with fenestration are detailed in Table 5. Of note, of the >1,100 cases of surgical fenestration and associated outcome reported in the studies, complications from fenestration were noted in only 15 cases (0.01%), suggesting that fenestration is a safe procedure and concern for surgical complication of various types may not be a valid reason for choosing not to fenestrate.

**Table 5:** Results of recent large-scale contemporaneous studies evaluating outcome and recurrence after hemilaminectomy with fenestration.

**Reference**	**Number of cases**	**Study design**	**Surgical approach**	**Recurrence rate**	**Fenestration-associated complications**	**Notes**
Mayhew et al. (72)	229	Retrospective case series	Hemilaminectomy without fenestration for all cases.	19.2%	Not applicable	None
Brisson et al. (34)	265	Retrospective case series	Hemilaminectomy for all cases. Prophylactic blade or power-assisted fenestration at various sites	4.4%	Pneumothorax (*n* = 1) Hemothorax (*n* = 1)	Hemothorax secondary to collagenase used for chemonucleolysis
Forterre et al. (69)	19	Prospective cohort	Hemilaminectomy for all cases. Single site power-assisted fenestration of the herniated disc vs. no fenestration	No recurrence in fenestrated group; 60% recurrence rate in non-fenestrated group although only 30% were clinical	None	Recurrence followed by MRI, not just clinical signs
Brisson et al. (66)	207	Prospective, randomized trial	Hemilaminectomy for all cases. Randomized to either single (herniated site only) or multi-site fenestration (T11-L4)	7.5% for multi-site fenestration and 18% for single site fenestration	Hemorrhage during fenestration (*n* = 7) Nerve root trauma (*n* = 4) Broken curette tip within the disc (*n* = 1)	Recurrence rate includes only confirmed recurrences; 92% of recurrences at a non-fenestrated site
Aikawa et al. (73)	662	Retrospective case series	Hemilaminectomy for all cases. Fenestration of all sites T11-12 to L1-2; L2-3 and L3-4 also performed in some dogs	2.3% based on clinical signs and imaging; 10% based on clinical signs alone	Intraoperative iatrogenic disc extrusion into the vertebral column (*n* = 1)	None

*Results are compared to recurrence rate noted by Mayhew et al. without fenestration*.

Several challenges exist in interpreting the existing literature on fenestration. First, there is no study that prospectively compares, in a randomized fashion, recurrence rate of IVDE in dogs that undergo hemilaminectomy with and without fenestration. As such, clinical demographic factors, surgeon preference and experience, fenestration technique, and other patient- and clinician- level factors likely bias the current literature. Developing a randomized trial to evaluate this question is difficult because veterinarians who fenestrate do so because they believe the literature supports that it is effective to reduce recurrence, and those who do not may not have experience or enthusiasm to do so if participating in a trial. Additionally, comparing recurrence rates between studies is difficult due to differences in outcomes monitoring and reporting. Some patients with a recurrence of IVDE may not return to a specialty care facility for a second incidence of clinical signs, or those with mild signs may be managed medically without confirmatory imaging. Thus, the true incidence and etiology of signs may be underestimated or unclear. To address this concern, longitudinal studies involving the owner could provide valuable data. Inclusion of only confirmed cases of IVDE recurrence in some studies, but not others, also limits comparison between techniques. Even so, the current literature supports prophylactic fenestration as a safe way to reduce future disc herniation at fenestrated sites. Published studies suggest that fenestration reduces the recurrence rate of IVDE, and surgeons should particularly consider fenestration of calcified discs. However, high quality evidence in support of this is not available and it is not known how many sites should be fenestrated to achieve the best outcome, nor is it known what is the effect of fenestration on development of IVDE in adjacent unfenestrated sites of substantial consequence (ex. L4-5 in the patient fenestrated from T11-L4).

## The Use of Neuroprotective Strategies

Various interventions have been evaluated in the context of acute spinal cord injury, many targeting secondary injury processes such as ischemia and vasospasm, inflammation, free radical production, ion channel disturbances and glutamate excitotoxicity.

### Methylprednisolone Sodium Succinate (MPSS)

The use of high dose steroids, particularly methylprednisolone sodium succinate, in acute spinal cord injury takes its roots from the experimental spinal cord injury literature, where the proposed mechanism of therapy was prevention of lipid peroxidation and secondary free radical injury (74–77). This therapy was evaluated in several high-profile human spinal cord injury trials which showed a potential small treatment effect when administered within 8 h after injury, although the results of those studies, and their clinical implications, remain controversial (78–81). Some experimental evidence, including a study in dogs, had also suggested that this therapy might be less useful than originally anticipated (82). In a recent prospective randomized placebo controlled blinded clinical trial evaluating the effect of MPSS on outcome in paraplegic deep pain negative dogs with IVDE, no treatment effect was observed with respect to locomotor recovery (83).

Many veterinary clinicians continue to use corticosteroids such as prednisone or dexamethasone routinely at lower, anti-inflammatory doses for the management of canine IVDE (26). The question of whether treatment with non-steroidal anti-inflammatories (NSAIDs) or steroids is most appropriate represents a somewhat polarizing issue in veterinary medicine and is highly clinician-dependent. One retrospective study demonstrated decreased odds of successful outcome with conservative therapy, and lower owner-reported quality of life scores, with the use of corticosteroids, irrespective of dose, duration, or specific steroid administered (23). While no study provides a prospective comparison of NSAIDs vs. anti-inflammatory doses of steroids in the management of IVDE in dogs, a recent study retrospectively evaluated clinical risk factors for the development of progressive myelomalacia in deep pain negative dogs and suggested that administration of corticosteroids may have a protective effect (62). The use of steroids at anti-inflammatory doses or NSAIDs in management of canine IVDE is an area where clinical equipoise exists and while controlling for pre-treatment of dogs prior to referral would present a challenge, this question could lend itself to prospective randomized trials evaluating outcome, quality of life, and incidence of myelomalacia between the two treatments.

### Polyethylene Glycol (PEG)

Polyethylene glycol (PEG) is a surfactant that gained popularity as a possible neuroprotective strategy for acute spinal cord injury. While not entirely understood, the proposed mechanism of PEG is that it acts as a fusogen to repair damaged neuronal cell membranes, prevent ion channel disturbances that lead to cytotoxic edema and secondary injury, and may also stimulate angiogenesis and promote axonal regeneration (84, 85). Studies in experimental models of SCI were encouraging for a positive treatment effect (86–88). Laverty et al. examined the therapy in a prospective open label canine clinical trial for dogs with IVDE (89). The canine study reported a positive treatment effect where a significantly higher number of deep pain negative dogs showed enhanced locomotor improvement but used a group of historical controls for which the recovery rate was less than typically reported for dogs who are deep pain negative secondary to IVDE. Subsequently, a prospective, placebo controlled, randomized, blinded trial evaluating the influence of PEG on outcome in deep pain negative dogs did not demonstrate a positive treatment effect (83).

### Other Strategies

A host of other neuroprotective strategies have been evaluated in both the laboratory and human clinical setting for treatment of acute spinal cord injury. Most promising based on positive findings in the experimental setting are minocycline, riluzole, and glybenclamide. At this time, there is no published efficacy data on any of these interventions for use in canine IVDE, although all three medications have known pharmacokinetics in the dog making them amenable to future veterinary clinical trials (90–92).

Several other adjunctive therapeutic strategies are not addressed in the present review but have been suggested throughout the veterinary literature. These interventions have only been evaluated in a small number of published studies, and include acupuncture, pulsed electromagnetic field therapy, chiropractic manipulation, and photobiomodulation (93–95).

## The Role of Physical Activity in The Development of and Recovery From IVDE

With respect to the role of physical activity in the development of and recovery from IVDE, several clinical questions exist regarding ideal daily activity levels for prevention of disease in dogs “at risk,” what constitutes appropriate restriction of activity after decompressive surgery, and the role of physiotherapy in post-operative recovery.

### Daily Activity for Dogs “At Risk” of IVDE

The influence of daily activity level on disc degeneration and the development of IVDE has been previously explored by Packer et al. who evaluated the impact of lifestyle on IVDE risk. It was observed that dogs receiving >1 h of daily exercise were less likely to have IVDE compared to dogs receiving <30 min of daily exercise and not allowed to jump on and off furniture (96). This study suggests that, while activity modification is often recommended by clinicians as a preventative measure against IVDE in chondrodystrophic breeds, this recommendation might be counter-productive.

### Post-operative Activity Restriction

Veterinary neurologists and surgeons tend to view strict activity restriction (also termed “cage rest”) as a vital component of both conservative and post-operative management of IVDE; however, the impact of this recommendation is poorly studied in dogs (26). Cage rest is usually defined as confinement to a small run or cage at all times except when the animal needs to eliminate. Cited goals of cage rest include allowing healing of the annulus fibrosus to prevent further extrusion of nucleus pulposus, prevention of further traumatic injury in an ataxic animal, and reducing pain and inflammation associated with affected nerve roots and meninges (25, 97–99). The duration of recommended cage rest is variable; however, some authors recommend as much as 6–8 weeks (26, 97). Whereas, long-term bed rest has not been shown to be beneficial in people with lumbar disc herniations, the argument for cage rest in the management of canine IVDE might be more logical based on anatomy, underlying pathophysiology, and inherent inability to reason with veterinary patients as to why they should consciously self-limit activity (100, 101). The influence of cage rest on outcome has been examined in only one published retrospective veterinary study in which outcome in dogs managed conservatively for IVDE was not influenced by duration of cage rest (23).

### Physiotherapy

An additional consideration with respect to activity modification is whether some forms of controlled activity, in the form of physiotherapy, might actually be beneficial to locomotor recovery in the postoperative setting, particularly in more severely affected dogs. This rationale stems from the experimental spinal cord injury literature, where a number of studies have demonstrated that intensive locomotor training can promote anatomic and physiologic changes within the injured spinal cord that might result in improved motor function (102–105). However, the experimental literature relating to physiotherapy is mixed, with persistent questions regarding timing and correct complement of activities to result in improved function vs. maladaptive neuroplasticity (106, 107). To a limited degree, some successful findings have translated to the human clinical setting where very small-scale open label studies have shown mild improvements in weight support, stepping, and spasticity with intensive locomotor training programs often coupled with cell-based therapies, or implantable epidural or nerve stimulation devices (108–112).

Various authors have advocated for the role of physiotherapy in canine IVDE, and physiotherapy is routinely recommended by many veterinary neurologists and orthopedic surgeons (26, 95, 113–115). Several studies have evaluated the role of physiotherapy in dogs with severe spinal cord injury caused by IVDE (93, 115, 116). Bennaim et al. conducted a prospective randomized controlled trial evaluating the influence of both physiotherapy and photobiomodulation on motor recovery in dogs with severe IVDE (non-ambulatory paraparesis or worse) (93). A positive treatment effect was not noted in that study, although the authors suggest that case numbers were not large enough to draw a firm conclusion. Conversely, a large-scale retrospective study of physiotherapy conducted by Jeong et al. evaluated neurologic outcome in dogs with IVDE causing injuries ranging from ambulatory paraparesis to paraplegic with absent nociception (115). The authors noted a significant improvement in locomotor outcome for dogs receiving surgical decompression coupled with physiotherapy when compared to those receiving surgical decompression alone. However, successful locomotor outcome for dogs with paraplegia with or without intact deep pain receiving decompressive surgery alone was only reported to be 17%, which is much lower than the typical outcomes reported in literature which range from 50 to 60%. Zidan et al. also conducted a prospective blinded trial where dogs with incomplete SCI caused by IVDE were randomized after surgical decompression to receive either a basic in-hospital physiotherapy program consisting only of passive range of motion and sling walking activities or a more intensive therapy program (116). No difference in locomotor outcome was observed between groups but this trial was designed to see if the rate of recovery of locomotion could be influenced in a population of non-ambulatory paraparetic and paraplegic deep pain positive dogs. It did not target dogs known to have less chance of recovery and suggests that a randomized controlled trial is indicated to investigate the influence of rehabilitation on recovery in dogs with more severe injuries that fail to show early improvement.

The current state of the veterinary literature does not support a role of routine physiotherapy to improve locomotor outcome in dogs with mild to moderate spinal cord injury caused by IVDE. A challenge in interpreting this literature is the fact that studies include dogs with all injury grades, many of which would assuredly recover with or without other intervention, making it difficult to demonstrate a treatment effect without a very large sample size. Additionally, there is not a standardized physiotherapy program followed across the field, making it is difficult to compare results between studies. Lastly, the type of physiotherapy shown to improve locomotor outcome in experimental injury models, and now employed in people with severe injuries, is a very intensive type of locomotor training. The types of activities described in these locomotor training protocols extend well-beyond the intensity of therapeutic activities implemented in veterinary medicine, which more classically includes under-water treadmill walking, passive range of motion exercises and assisted weight-supported walking. It should also be noted that the primary aim in the acute phase of IVDE is to regain movement, over-ground locomotion and balance but “under-water” treadmill is best suited to improve strength and muscle mass. Activities directed toward restoration of movement, such as the use of “over-ground” treadmill training (as shown in people to improve motor recovery and balance (*e.g.*, using proprioceptive platforms) may actually be more rational early on in the recovery process for IVDE and should be encouraged first (117).

In people with severe spinal cord injury, more intensive protocols are often coupled with external or implantable assistive devices not used in veterinary medicine. For example, robotic assisted gait training clearly reduces spasticity and improves lower limbs motor function in people with severe spinal cord injury (118). Thus, findings from the human clinical setting may not reflect those reasonably expected in veterinary medicine using current approaches or previously evaluated patient populations. A recently published retrospective study by Gallucci et al. showed an increase in the development of “spinal walking” in deep pain negative dogs despite the fact that these dogs did not regain pain perception (119). Further prospective studies are needed to determine what impact intensive physiotherapy may have in deep pain negative dogs, or on parameters beyond locomotor recovery including important SCI comorbidities such as pressure sores, neuropathic pain, and spasticity. In that respect, consensus about retraining after spinal cord injury lags far behind what is applied in humans where the literature covering that topic is vast. In particular, recent reviews and meta-analysis have clearly shown the benefit of several physiotherapy interventions to improve voluntary muscle strength (120). This includes interventions such as resistance training, functional electrical stimulation or robotic gait training. Some of these are difficult to implement in dogs because of cost and the challenges of eliciting specific voluntary movements on command, but others such as functional electrical stimulation, “over-ground” treadmill training and proprioceptive platform training are more feasible.

## Conclusions and Recommendations

There exist a number of gaps in the veterinary literature with respect to standards of care for dogs with acute thoracolumbar IVDE. Areas identified for future study include comparison of medical and surgical management for dogs with more mild signs associated with IVDE (those who are only painful or have ambulatory paraparesis), the effect of early decompression in locomotor recovery in dogs with non-ambulatory paraparesis or paraplegic with intact deep pain, and on the incidence of progressive myelomalacia in deep pain negative dogs, the effect of durotomy coupled with spinal decompression in dogs with severe injuries, and the influence of intensive physiotherapy in deep pain negative dogs. Recommendations for standard care can be made in some areas, particularly with respect to surgical decompression where the currently available evidence supports that surgery should be recommended for dogs with non-ambulatory paraparesis or worse. While additional information is needed about the influence on timing of decompression on outcome in dogs that are deep pain negative for longer than 48 h duration, there is no evidence to support treatment of the 48 h time point as a cut off beyond which it becomes impossible for dogs to achieve locomotor recovery. Surgical decompression is best accomplished by either hemilaminectomy or mini-hemilaminectomy and fenestration of, at a minimum, the acutely ruptured disc. Adjacent discs easily accessed by way of the same approach should be considered for fenestration given the evidence that this substantially reduces future herniation at fenestrated sites. Currently available neuroprotective strategies such as high does MPSS and PEG are not recommended due to lack of demonstrated treatment effect in randomized controlled trials. The role of anti-inflammatory steroids as a protective strategy against progressive myelomalacia and the question of whether anti-inflammatory steroids or NSAIDs provide superior medical therapy require further evaluation.

## Author Contributions

SM, AT, NO, VS, and NG conceived, wrote, and edited the manuscript. CANSORT-SCI edited the manuscript. All authors have approved the final submitted version.

## Conflict of Interest

The authors declare that the research was conducted in the absence of any commercial or financial relationships that could be construed as a potential conflict of interest. The handling Editor declared a shared affiliation with one of the authors NG.
